# Molecular dynamics simulation of the brain-isolated single-domain antibody/nanobody from camels through *in vivo* phage display screening

**DOI:** 10.3389/fmolb.2024.1414119

**Published:** 2024-09-02

**Authors:** Behnam Hasannejad-Asl, Hassan Hashemzadeh, Farkhondeh Pooresmaeil, Mehran Dabiri, Mohammad-Reza Pooresmaeil, Davoud Ahmadvand, Arshad Hosseini

**Affiliations:** ^1^ Department of Medical Biotechnology, School of Allied Medicical Sciences, Iran University of Medical Science, Tehran, Iran; ^2^ Department of chemistry, University of Birjand, Birjand, Iran; ^3^ School of Biomedical Engineering, Faculty of Engineering and IT, University of Technology Sydney, Sydney, Australia; ^4^ Medical education research center, Guilan University of Medical Sciences, Rasht, Iran; ^5^ Department of Medical Laboratory Sciences, Faculty of Allied Medicine, Iran University of Medical Sciences, Tehran, Iran; ^6^ Neuroscience Research Center, Iran University of Medical Science, Tehran, Iran

**Keywords:** blood-brain barrier, drug delivery, Camelus single-domain antibody, phage display library, molecular docking, molecular dynamics simulation

## Abstract

**Introduction:**

During the last decade, there has been a significant rise in the use of therapeutic antibodies or passive immunotherapy for treating various conditions like inflammation and cancer. However, these proteins face challenges reaching the brain and often require specialized delivery methods such as single-domain antibodies (sdAbs). Traditional antibodies struggle to efficiently cross the blood-brain barrier (BBB), hindering their effectiveness. Receptor-mediated transcytosis (RMT) offers a promising pathway for transporting large molecules essential for brain function and treatment across the BBB.

**Methods:**

SdAbs and peptide ligands with an affinity for RMT receptors are commonly employed to enhance the transport of biotherapeutics compounds across the BBB. This research used a sdAbs phage-displayed library from 13 *camelus dromedarius* samples to identify sdABs that specifically bind to and are internalized by human BBB endothelial cells (ECs) through *in vivo* panning.

**Results and discussion:**

One sdAb, defined as FB24, was isolated, sequenced, translated into an open reading frame (ORF), and subjected to three-dimensional (3D) modeling. Molecular docking and molecular dynamics simulations were carried out by the HADDOCK web server and GROMACS, respectively, to evaluate the interaction between FB24 and EC receptors *in silico*. The docking results revealed that FB24 exhibited binding activity against potential EC receptors with −1.7 to −2.7 ranged z score and maintained a stable structure. The docked complex of FB24-RAGE (receptor for advanced glycation end products, also known as advanced glycation end product receptor [AGER]) showed 18 hydrogen bonds and 213 non-bonded contacts. It was chosen for further analysis by molecular dynamics simulations by GROMACS. This complex showed a stable condition, and its root mean square deviation (RMSD) was 0.218 nm. The results suggest that FB24 could serve as a suitable carrier vector for transporting therapeutic and diagnostic agents across the BBB to the brain through a non-invasive route.

## Graphical Abstract

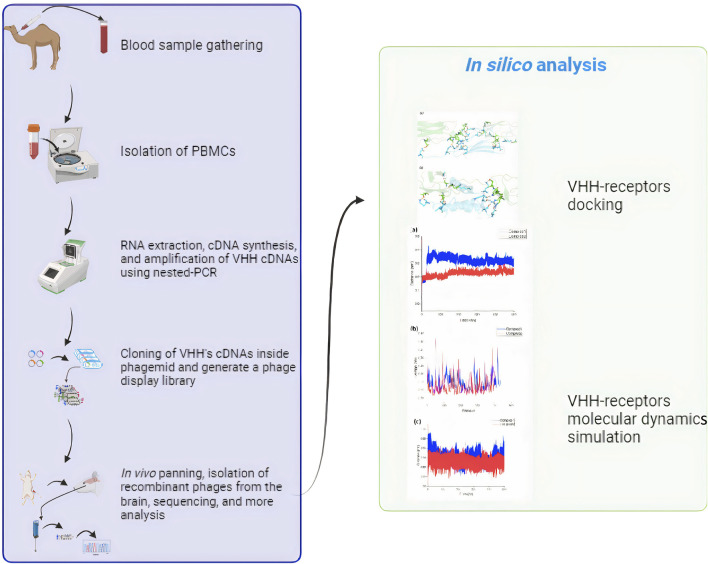

## 1 Introduction

Despite significant advancements in neuroscience and drug research, the blood-brain barrier’s (BBB) limited permeability has consistently challenged the effectiveness of several promising drugs for treating disorders affecting the central nervous system (CNS) (Georgieva et al., 2014; Pardridge, 2017). The CNS’s access to therapies delivered peripherally is significantly restricted by the BBB (Hasannejad-Asl et al., 2023). The BBB’s endothelial cells (ECs) have tight connections prohibiting most synthetic compounds and almost all macromolecules, including peptides and biologics, from being transported paracellularly (Pardridge, 1995). In particular, for recently developed classes of antibodies and therapeutic proteins, reaching brain targets remains a significant problem in establishing treatments for neurological disorders.

Multiple transport channels are available via the BBB to facilitate the brain’s provision of necessary proteins and nutrients (Power et al., 2022). Several of these transport channels can be utilized for the transportation of pharmaceutical molecules (Hasannejadasl et al., 2020). The physiological process of receptor-mediated transcytosis (RMT) is crucial to transendothelial biologic delivery. In this process, particular receptors of BBB such as transferrin receptor (TfR) (Johnsen et al., 2019), insulin-like growth factor 1 receptor (IGF-1R) (Vewinger et al., 2019), Receptor for Advanced Glycation End products (RAGE) (also known as advanced glycosylation end product receptor (AGER)) (Anthony et al., 2021), and low-density lipoprotein receptor protein 1 (LRP1) (Broadwell and Banks, 1993), among others, are activated by the binding of ligands to facilitate internalization and transport through brain endothelial cells (BEC), and in certain instances, to release the ligand cargo on the outer surface of the BBB. Concentrating on these endogenous transcytosis processes is one of the most promising methods for creating a non-invasive, secure, and targeted cross-BBB biologic delivery system. A possible method is the creation of specialized antibodies that target the RMT system, precisely targeting BBB receptors and transporting them into the brain in a regulated and non-harmful way as a biological “Trojan Horse” delivering medicinal substances (Patel et al., 2009; Soni et al., 2010; Pardridge and Boado, 2012) The effectiveness of this approach for delivering drugs to the CNS has already been thoroughly confirmed for two specific targets: the insulin receptor (IR) and the transferrin receptor (TfR). Antibodies to these two receptors have shown the ability to transport therapeutic medicines over the BBB via RMT (Pardridge, 2012; Salvati et al., 2013; Boado and Pardridge, 2017), supporting th promise of this route for the treatment and diagnostics of neurological disorders. Apart from these EC receptors, the RAGE also interacts with many polypeptide ligands, such as HMGB1 (also known as amphoterin), S100/calgranulins family of polypeptides (e.g., S100B, S100P, S100A1, S100A2, S100A4, S100A5, S100A6, S100A7, S100A8/A9, S100A12 and S100A13) (Leclerc et al., 2009; Ramasamy et al., 2009; Koch et al., 2010), macrophage-1 antigen (Mac-1) (Pullerits et al., 2006), and amyloid-β toxins (Aβ) (Sturchler et al., 2008; Liu et al., 2010) and therapeutic anti-RAGE antibodies (Deane et al., 2012).

Among the most significant biopharmaceutical compounds with a substantial market share are antibodies. Clinical therapies utilizing antibodies are widely used for a variety of human disorders, including CNS diseases like Parkinson’s disease (Feng et al., 2018), multiple sclerosis (Di Pauli and Berger, 2018), amyotrophic lateral sclerosis (Coban et al., 2013) and Alzheimer’s dementia (Moreth et al., 2013; Decourt et al., 2017). However, their biodistribution and delivery strategies still require improvement, as their size and poor tissue penetration are their limitations (Soleimanizadeh et al., 2021). Because of their smaller size, derivatives like single-domain antibodies (sdAbs), also called VHH, and Nanobodies (Nbs) are becoming increasingly popular for therapeutic and clinical diagnostics. Compared to traditional antibodies, smaller antibodies may be produced at lower prices, providing several advantages for therapeutic applications. The smallest known antibody fragments with binding functions are called Nbs, and they are heavy-chain variable region fragments discovered in sharks and camels (Muyldermans, 2013; Banihashemi et al., 2018; Banihashemi et al., 2021). These fragments possess favorable attributes, including low molecular weight, high tissue penetration capability, and straightforward preparation. As a result, they have been extensively employed in both scientific studies and therapeutic applications. Examples of these include the diagnosis of infections, (De Vlieger et al., 2018), utilization as probes in biosensors (Dupré et al., 2019) and treating conditions like cancer and inflammation. (Van Audenhove and Gettemans, 2016).

Nowadays, in addition to wet lab investigations, computational therapeutic compound discovery can accelerate the challenging process of designing and optimizing a new drug candidate (Jorgensen, 2004). The impact of computational structure-based drug design (SBDD) on drug discovery has intensified in the last decade (De Vivo, 2011). Molecular docking is a critical computational approach that simulates the interactions between ligands and their receptors. The process of molecules being recognized by the receptor and ligand involves various intermolecular interactions, such as hydrogen bond activities, electrostatic reactions, Van der Waals contacts, etc. (Choupani et al., 2022). By employing a computational approach, molecular docking may anticipate the binding affinity and mechanism of action of ligands or drugs, making it useful for the virtual screening of ligands or drugs. MD simulation and associated techniques are also on the way to becoming standard computational tools in drug research. Their primary benefit lies in their explicit handling of entropic effects and structural flexibility. When more sophisticated algorithms and hardware designs are used, this enables more precise estimation of the thermodynamics and kinetics related to ligand-target recognition and binding (De Vivo et al., 2016).

In recent study, a diverse nanobody phage display (PD) library was created from 13 naïve Camelus dromedaries, leading to the isolation of a specific VHH capable of crossing the BBB in rats. This VHH, known as FB24, was subsequently subjected to physiochemical analysis, three-dimensional (3D) structural modeling, and prediction of its transfer pathway using peptide-protein docking and MD simulation. The findings revealed that FB24 interacts with BBB receptors, particularly RAGE, with high affinity, suggesting that RAGE is the most probable receptor for FB24 binding and BBB penetration.

## 2 Material and methods

### 2.1 Materials

The Wistar rats used in this study were sourced from the Pasteur Institute of Iran. The necessary equipment such as the cellular RNA extraction kit, Gel extraction DNA kit, and bacteria culture media were procured from reputable suppliers: Roche, Biofact, and GIBCO-BRL. Chemical reagents including HEPES, various salts, tween-20, and dextran were acquired from Sigma Aldrich. Specialized items such as nested PCR primers, M13 helper phage, pComb3XSS phagemid, and Er2738 bacteria were generously provided by Dr. Fatemeh Rahbarizadeh from Tarbiat Modarres University, Tehran, Iran. Additionally, all other biochemical and molecular biology reagents were obtained from a variety of trusted companies.

### 2.2 Construction of camelid sdAB PD library

#### 2.2.1 Peripheral blood mono nuclear cell (PBMC) gathering

Thirteen camel blood samples were collected from non-immunized camels, and 2 mg/mL EDTA was added to prevent blood clotting. The PBMC was isolated using a Ficoll gradient at a ratio of 2:1, then centrifuged at 400 *g* for 30 min at 20°C and resuspended in PBS buffer. The purified cells were washed twice with PBS buffer, divided into RNAase-free microtubes, and stored at −70°C.

#### 2.2.2 VHH’s cDNA amplification and construction of VHH-pComp3xss recombinant phagemids

The process of amplifying VHH’s cDNA and constructing VHH-pComp3xss recombinant phagemids has been previously described in detail (Ismail et al., 2021). Briefly, a total RNA of 20 × $10^{9}$ PBMCs was extracted using a High Pure RNA Isolation Kit (Roche, Germany) following the manufacturer’s instructions. Subsequently, cDNA was synthesized using a reverse transcription kit (Yekta tajhiz, Iran). Nested polymerase chain reaction (PCR) was utilized for VHH’s cDNA amplification. During the first round, 6 primer pairs were used to separate classical VH and VHH cDNAs. (Table 1). The VHH cDNA band (600 bp) from the first round PCR product was extracted from the 1% agarose gel using BioFact™ PCR Purification Kit (Biofact, South Korea) and used as a template for the next round of PCR. The second round of PCR employed primer pairs containing sfiI restriction sites (Table 2). The PCR products were run on agarose gel, purified, digested with sfiI, and then ligated into linearized pComb3XSS phagemid using T4 DNA ligase. The PCR solution involved 10 picomol of each primer, deionized water, 200 μ molar of various dNTPs, and 0.5 U/μL DNA Taq polymerase. Each round of nested PCR had the same conditions involving primary denaturation at 94°C for 5 min, cycles of denaturation at 94°C for 20 s, annealing 55°C for 40 s, extension at 72°C for 40 s and final extension at 72°C for 8 min. PCR solution included primers, deionized water, various dNTPs, and DNA Taq polymerase.

**TABLE 1:** First round PCR primer pairs.

Primers	Sequence
Calloo1 (for)	GTC CTG GCT CTC TTC TAC AAG G
Calloo2 (rev)	GGT ACG TGC TGT TGA ACT GTT CC
M for1 (for)	CTG TTC CTC CTT TGG CTT CGT GTT
M back1 (rev)	TGG GTG GTC CTG GCT GCT CTT C
Mit for (for)	ATG GAG AGG ACG TCC TTG GGT
Mit rev (rev)	TTC GGG GGG AAG AGR AAG AC
FPF-MJ (for)	GCC CAG CCG GCC ATG GTA AAG CTG GAG TCT
CH2B3 (rev)	GGG GTA CCT GTC ATC CAC GGA CCA GCT GA
FPF-MJ2 (for)	GCC CAG CCG GCC ATG GCC CAG GAG GAG TCT GGG
CH2 For A4 (rev)	CGC CAT CAA GGT ACC AGT TGA
FPF-MJ3 (for)	GCC CAG CCG GCC ATG GCC CAG GCT CAG CTG GTG GAG TCT
CH2B3 (rev)	GGG GTA CCT GTC ATC CAC GGA CCA GCT GA

Abbreviations: for: forward primer; rev: reverse primer.

**TABLE 2:** Second round PCR primer pairs.

Primers	Sequence
MAAH (for)	ACT GGC CGG CCT GGC CTG AGG AGA CGG TGA CCT G
MMA (rev)	ACT GGC CCA GGC CSA GGT SCA GCT CSW GGA G
Back for (for)	CGT GGC CCA GGC GGC GGA GTC TGG RGG AGG
For Back (rev)	TGC GGC CGC TGG AGA CGG CCG GCC TGG CCT GGG T
Back A4 (for)	CGT GGC CCA GGC GGC CAT GGC CGA KGT SCA GCT
MMA (rev)	ACT GGC CGG CCT GGC CTG AGG AGA CGG TGA CCT G
Llama Alpha 1 (for)	CGT GGC CCA GGC GGC CCA GGA KGT SCA GCT
MMA (rev)	ACT GGC CGG CCT GGC CTG AGG AGA CGG TGA CCT G

Abbreviations: for: forward primer; rev: reverse primer.

#### 2.2.3 Preparation of electrocompetent bacteria

ER2738 bacteria were utilized for the amplification of recombinant phagemids in our experiment. The bacteria were initially grown on LB agar overnight, followed by the transfer of a single colony to LB broth medium for further incubation at 37°C and 250 rpm overnight. Subsequently, a portion of this cultured bacteria (10 mL) was added to 500 mL of fresh Super Broth (SB) medium and allowed to grow at 37° with continuous shaking until it reached the logarithmic growth phase. The bacteria were then transferred to a sterile tube placed on ice and centrifuged at 4,000 *g* at 4°C. The supernatant was discarded, and the pellet was washed with cold sterile distilled water in three consecutive steps: 40 mL, 20 mL, and 10 mL, followed by centrifugation under the same conditions.

#### 2.2.4 Transfect the recombinant phagemids into competent bacteria

To introduce the recombinant phagemids into competent bacteria, electrocompetent ER2738 bacteria were electroporated with the pComb3XSS-VHH phagemids using 2,500 V electroporation for 5 ms. The electroporated bacteria were then recovered in 2 mL of SOC medium containing 2% glucose and were incubated at 37°C for 1 h.

Subsequently, ampicillin was added to the culture medium, and the bacteria were further incubated overnight at 37°C. To confirm the successful incorporation of VHH into the vector, 105 colony-forming units of transformed bacteria were cultured on LB agar, and ten colonies were randomly selected for colony PCR using specific primers (forward, 5′-GCCCCCTTAGCGTTTGCCATC-3′, and reverse 5′AAGACAGCTATCGCGTTTGCCATC-3′). The PCR conditions were consistent with those mentioned in section 2.2.2.

### 2.3 Rescue of phage library

As mentioned previously, the single-domain antibody (sdAb)-displaying phages were obtained through phage rescue with the use of M13 K07 helper phage, along with Kanamycin as a selection marker (Singh et al., 2010). In this process, the genetically modified bacteria were grown in SB medium containing 0.1% glucose. The M13 helper phage was then introduced to the culture and allowed to incubate at 37°C for half an hour. Subsequently, Kanamycin was added to the mixture, shaken, and further incubated at 37°C for an additional hour. The next step involved centrifugation at 3,500 rpm for 10 min, after which the pellet was resuspended in SB medium and left to shake at 37°C overnight. To isolate the recombinant phages from the bacterial culture, the solution underwent centrifugation at 4,000 g for 20 min at 4°C. The resulting supernatant was then transferred to microtubes, followed by the addition of a Polyethylene glycol/NaCl (PEG/NaCl) solution in a 5:1 ratio. This mixture was incubated on ice for an hour before being subjected to further centrifugation at 19,000 *g* for 30 min at 4°C. The resulting pellet containing the phages was then resuspended in 4% milk solution in Tris-buffered saline (TBS) and used for phage titration. For the phage titration process, a series of dilutions were prepared. The diluted phages were then added to logarithmically growing bacteria and incubated at 37°C for 30 min. The infected bacteria were subsequently plated on TOP agar and left to incubate at 37°C overnight for further assessment.

### 2.4 Conducting phage library panning in vivo

During the *in vivo* panning of the phage display library, all procedures involving handling of animals were carried out in accordance with the guidelines provided by the Institutional Review Board (IR) and were approved by the Animal Care and Ethical Committee with reference number IR.IUMS.FMD.REC 1396.9511522006. The animals used in the study were of similar weight and were housed in a temperature and humidity-controlled room with a 12-h light and 12-h dark cycle. For the *in vivo* panning process, a total of 1.7 × 10^13 plaque-forming units (pfu) of recombinant phages were intravenously injected into 3 Wistar rats. After a period of 6 h, the animals were anesthetized using a combination of ketamine and xylazine, following which they were perfused with 200 mL of 99.99% physiological serum containing 0.2% tween-20. Once the perfusion was completed and all the blood was cleared from the vessels, the brain tissue was carefully extracted and placed in a 0.8 mL physiological buffer containing HEPES (2-[4-(2-hydroxyethyl)piperazin-1-yl]ethanesulfonic acid or 4-(2-Hydroxyethyl)piperazine-1-ethanesulfonic acid or N-[2-Hydroxyethyl]piperazine-N′-[2-ethanesulfonic acid]), NaCl (Sodium chloride), KCl (Potassium chloride), CaCl_2_ (Calcium dichloride), MgCl_2_ (Magnesium dichloride), NaH_2_PO_4_ (Monosodium phosphate), glucose, and dextran. Dextran was utilized to remove the blood vessels to just brain tissue remains for the subsequent investigations. The brain tissue was then homogenized at 4°C and centrifuged at the same temperature and a speed of 5,400 g for 15 min. Following centrifugation, 100 μL of the supernatant was mixed with logarithmic vector-free bacteria and incubated at room temperature for 45 min. Subsequently, ampicillin was added to the culture media of the bacteria and further incubated at 37°C for an additional 30 min. Finally, 20 μL of the surviving bacteria that had been exposed to the phages were cultured on selective TOP agar containing ampicillin. The *in vivo* panning process was repeated for three rounds, utilizing the phages isolated in the preceding step each time. Rats that were intravenously injected with non-recombinant phages served as the negative control.

### 2.5 Colony PCR and sequencing

Following each round of panning, five plaques were randomly selected as templates for a PCR reaction using general primers. The PCR conditions and solutions were consistent with those previously described for nested PCR, and the results were run on a 1% agarose gel. Additionally, ten colonies were randomly chosen after each panning round for Sanger sequencing to confirm the identification of recombinant phages isolated from the brain tissue.

### 2.6 Physiochemical analysis of isolated VHH

Analysis of the physiochemical properties of the isolated VHH (FB24) was conducted, which included parameters such as molecular weight (MW), theoretical isoelectric point (pI), total number of negative and positive charged residues, and Grand average of hydropathicity (GRAVY). This analysis was performed using the protparam tool available at https://web.expasy.org/protparam/(Gasteiger et al., 2005; Walker, 2005). Additionally, the secondary structure of the isolated VHH was predicted using the PSSpred tool, accessible at https://zhanggroup.org/PSSpred/(Yan et al., 2013). This tool utilizes a neural network training algorithm to accurately predict protein secondary structures.

### 2.7 Forecasting and validation of the unique VHH 3D structure

The 3D structure of brain FB24 was forecasted utilizing the I-TASSER (https://zhanggroup.org/I-TASSER/) (Yang et al., 2015), trRosetta (https://yanglab.qd.sdu.edu.cn/trRosetta/) (Du et al., 2021), and RaptorX online servers (http://raptorx6.uchicago.edu/) (Wang et al., 2017). To assess the accuracy of the predicted 3D structure, Verify3D (Lüthy et al., 1992) and PROCHECK (Laskowski et al., 1996) modules of SAVES v6.1 (https://www.doe-mbi.ucla.edu/verify3d/) (Bowie et al., 1991) were utilized. These tools were employed to determine how well the 3D model matched with its amino acid sequence and to display the Ramachandran Plot in relation to phi-psi probabilities. The structure’s quality was confirmed based on various factors including the percentage of residues in specific areas, the number of proline and glycine residues, and the orientation of dihedral angles such as psi and phi, as well as backbone conformation.

### 2.8 Fetching BBB endothelial cell receptor 3D structures

Ectodomains of most expressed BBB EC receptors, such as RAGE, TfR1, LRP1, and IGF-1R, were fetched from the Protein Data Bank (PDB) (https://www.rcsb.org/). Based on the resolution and the complexes present in the crystal structures, the most appropriate structures of the receptors [RAGE, PDB: 4P2Y (2.3 Å), TFR1, PDB: 6WRV (2.47 Å), IGF-1R, PDB: 5U8Q (3.27 Å), LRP1, PDB: 1N7D (3.7 Å)] were chosen for optimization and protein-protein docking.

### 2.9 Peptide-protein docking

Peptide-protein docking involved several steps to ensure accurate results. Firstly, the optimization of FB24, crystal ligands, and receptor structures was carried out. Nonstandard residues, complexed peptides, and solvents were removed while hydrogen and charges were added, and side chains were completed using Chimera 1.15src software (Pettersen et al., 2004).

The prepared structures then underwent energy minimization for 50,000 steps. Subsequently, they were equilibrated in both NVT and NPT ensembles for 2 ns and 5 ns, respectively. MD simulation was conducted for 5 ns using the GROMACS version 2023.3 (Páll et al., 2015). It should be noted that all force field parameters are obtained from charmm36 force field (Gutiérrez et al., 2016).

The binding residues of receptors were identified from the complex structures. The 3D structures of proteins can be found in Supplementary Table S1. Peptide-protein docking was performed using HADDOCK (https://wenmr.science.uu.nl/haddock2.4/) (Honorato et al., 2021), an online web server. Complexed peptides from each receptor served as positive controls (RAGE: S100A6; TFR1: Computationally designed protein 3DS18; IGF-1R: IGF-1; LRP1: PCSK9).

### 2.10 Utilizing molecular dynamics simulations

Based on peptide-protein docking results, the FB24-RAGE and its crystal structure (S100A6-RAGE) were selected for further analysis by MD simulations. The MD simulations were conducted using the GROMACS 2023.3 software with the charmm36 force field. In each complex, the peptide-protein complex was placed centrally within the simulation box, positioned 1.0 nm away from the box walls with periodic boundary conditions (pbc) implemented. The system was then solvated with TIP3P water molecules. To achieve a neutral solvation box, Na^+^ and Cl^−^ ions were added at a concentration of 0.15 M. The system’s energy was minimized through the steepest descent method with 50,000 steps.

To maintain a stable environment, temperature and pressure were coupled using Nose-Hoover and Parinello-Rahman methods, respectively, at 310 K and 1 bar. Temperature and pressure couplings were set at 0.1 and 2.0 ps, respectively. The Partial Mesh Ewald (PME) method was employed for the calculation of van der Waals (vdW) and electrostatic interactions. Cut-off distances for vdW and Coulomb interactions were set to 12 Ǻ, with a neighbor list of 12 Ǻ as well. Bond lengths were constrained using the linear constraint solver (LINCS) algorithm, while a time step of 2 fs was implemented. The system was equilibrated for 5 ns under both NVT and NPT conditions before conducting a 500 ns MD simulation. Trajectories were saved every 2 ps for further analysis.

Analysis of the simulations was carried out using the internal GROMACS modules and Xmgrace software. Parameters such as Root Mean Square Deviation (RMSD), Root Mean Square Fluctuation (RMSF), and Radius of Gyration (Rg) were assessed to understand the dynamics of the peptide-receptor complex.

## 3 Results

### 3.1 Isolation of lymphocytes and cDNA synthesis

Lymphocytes from dromedary camels were separated using a Ficoll density gradient of 1,231 g/mL. The process involved harvesting cells with a yield ranging from 57.9% to 99% PBMC, from which mRNA molecules were extracted. Subsequently, a cDNA library was constructed from the isolated mRNA using poly-T primers.

### 3.2 Construction of nanobodies PD library and phage titration

**FIGURE 1:**
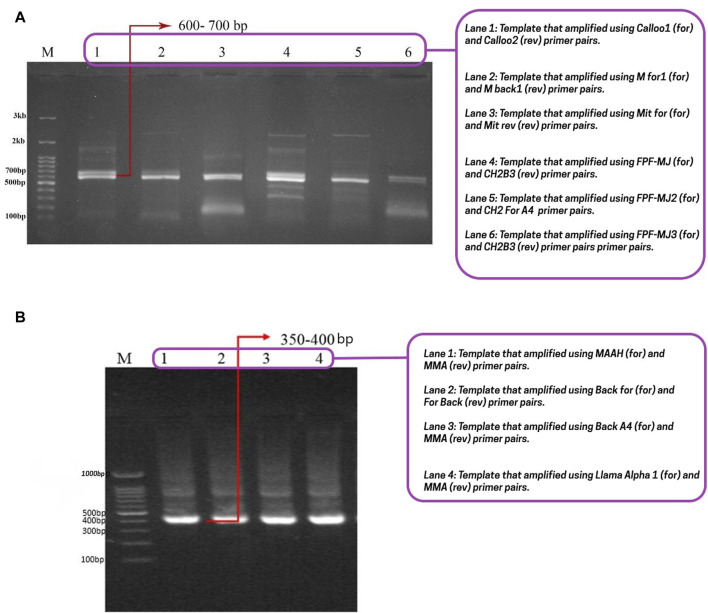
Conventional heavy chain, camelid antibody’s heavy chain, and nanobody gel. **(A)** The first step of nested-PCR involves amplifying the conventional heavy chain (850–900 bp) and the heavy chain of camelid antibodies (600–700 bp). The lanes 1, 2, 3, 4, 5, and 6 correspond to the templates amplified with Calloo1 (for) and Calloo2 (rev), M for1 (for) and M back1 (rev), Mit for (for) and Mit rev (rev), FPF-MJ (for) and CH2B3 (rev), FPF-MJ2 (for) and CH2 For A4 (rev), and FPF-MJ3 (for) and CH2B3 (rev) primer pairs, respectively; **(B)** the second step of nested-PCR that shows nanobody or sd-Ab binding at 350–400 bp. The lanes 1, 2, 3, and 4 correspond to the templates that were amplified with the MAAH (for) and MMA (rev), Back for (for) and For Back (rev), Back A4 (for) and MMA (rev), and Llama Alpha 1 (for) and MMA (rev) primer pairs, respectively. Abbreviations: For: Forward; Rev: Reverse.

After creating a naive library, our primary goal was to identify the most effective nanobodies for transfer through the BBB. Initially, the heavy chain of a conventional antibody (900 bp) and a camelid antibody (600–700 bp) were amplified (Figure 1A). Then, in the second step of PCR, VHH specific primers and a 600–700 bp DNA band served as templates, resulting in the amplification of camelid single-domain antibodies (VHH: 350–400 bp) (Figure 1B). Subsequently, the VHH regions were inserted into the pCom3XSS phagemid vector to develop a versatile PD library.

The number of plaques present in the gradient dilution tubes was counted to determine the titer of this nanobody library. By incorporating the VHHs into the pComb3Xss phagemid vector, a phage antibody library with a titration of approximately 5 × 10^11^ PFU/mL was established, showcasing a high likelihood of obtaining nanobodies with precise specificity and diverse sequences. Random colonies were chosen for PCR analysis, revealing a 100% insertion rate within the library.

These findings show that a successful, high-quality PD library was built to choose BBB transferrable nanobodies. Phage titer was calculated as follows:

Phage titration (plaque number (pfu) × Dilution Factor (μL/mL)/V ×D.

V is the volume of diluted phage added to the bacteria culture, and D is the phage dilution.

### 3.3 Exploration of PD library through *in vivo* panning

PD is a potent technique for determining an antigen with good receptor-binding affinity. It has been argued that *in vivo* PD screening methods have some benefits compared with *in vitro* selection procedures because VHHs expressed on the surface of phages can be selected in the complicated environment of the animal body (Pettersen et al., 2004). Nanobodies are determined and analyzed functionally, and have to defeat intricate obstacles and degradation mechanisms.

The blood-brain barrier (BBB) endothelial cells are influenced by surrounding cells and the blood flow within the vessels. This complexity of the BBB results in the regulation of specific receptors on the cell surface in a polarized manner. This indicates that the selection of highly effective VHHs capable of crossing the BBB should ideally be done *in vivo*.

Therefore, in order to identify camel-derived brain-targeting nanobodies and conduct a computational analysis of screened molecules, we carried out an *in vivo* PD selection approach as detailed in the materials and methods section. In summary, the PD library was injected intravenously into rats. After 6 h, the animals were euthanized, their brains were harvested, and phages were isolated to capture brain-specific VHHs.

The time point for sampling was selected based on preliminary experiments to optimize the identification of VHHs that rapidly localize to the brain. Three rounds of selective screening were performed to enrich the population of brain-targeting VHHs. At the conclusion of the third round, the recovered phages displayed a titer of 10^3^ phages/mL, indicating an enhanced ability to reach the BBB. Conversely, no phages were isolated when non-recombinant phages were used as a control. In conclusion, the *in vivo* PD approach successfully identified VHHs that target the BBB with high specificity and efficiency.

### 3.4 Sequencing of VHHs isolated from the brain

**FIGURE 2:**
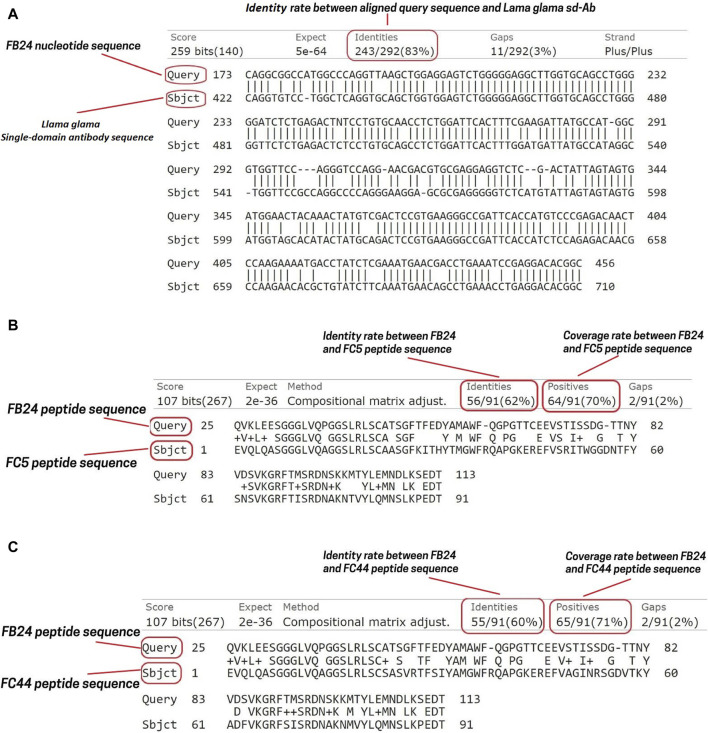
FB24 nucleotide and amino acid sequence alignment. The nucleotide alignment of FB24 against llama and *Camelus dromedarius* resulted in 100 hits. **(A)** The terms “Query” and “Subject” correspond to the FB24 and Llama glama immunoglobulin heavy chain variable domain (VHH2) nucleotide sequences, respectively. This alignment indicates that FB24 shares 83% identity with the *Lama glama* immunoglobulin heavy chain variable domain (VHH2); **(B)** FB24 BLASTP analysis against FC5 revealed 62% identity and 70% coverage; **(C)** FB24 BLASTP analysis against FC44 showed 60% identity and 71% coverage.

Phages isolated from the latest *in vivo* panning of the brain were sequenced utilizing the Sanger sequencing technique. This method of DNA sequencing relies on the random attachment of dideoxynucleotides (ddNTP) by DNA polymerase during the replication of DNA *in vitro*. Subsequently, the sequence was compared against the nucleotide collection of camelus dromedaries and llama organisms using the nucleotide blast tool of the National Center for Biotechnology Information (https://blast.ncbi.nlm.nih.gov/Blast.cgi). The BLASTN results revealed 100 hits, with the immunoglobulin heavy chain variable domain of the llama glama showing 83% Identity, 62% query coverage, and an E-value of 7e-68 (Figure 2A). The nucleotide sequence was then translated into the peptide sequence of the open reading frame (ORF) using the ORF finder server (https://www.ncbi.nlm.nih.gov/orffinder/). Subsequently, the ORFs of varying lengths ranging from 13 to 275 residues were aligned using BLASTP, and the most closely related peptide to camel antibodies was designated as VHH FB24. Additionally, FB24 aligned with VHHs FC5 and FC44 confirmed as BBB transfer camelid VHHs. BLASTP results indicated that FB24 shares 62.00% and 60.00% Identity with FC5 and FC44, respectively, while the coverage with both FC5 and FC44 is 78% (Figures 2B, C).

### 3.5 *In silico* analysis of FB24 physiochemical characteristics

Analysis of the nucleotide sequence revealed that the brain-isolated FB24 encodes a 113 amino acid peptide. Physiochemical properties of FB24 were predicted using Protparam online web server (https://web.expasy.org/protparam/) (Gasteiger et al., 2005; Walker, 2005) and demonstrated that FB24 has a molecular weight of 11.98 KD, with a pI of 4.82. The estimated half-life of FB24 in mammalian, yeast, and *Escherichia coli* cells is 30 h, >20 h, and >10 h, respectively. There are 10 positive residues (Arg and Lys) and 13 negative charged residues (Asp and Glu) in FB24. Further properties are detailed in Table 3. The secondary structure of FB24 consists of 15% α-helices, 39% β-sheets, and the remaining structure comprises coils and turns.

**TABLE 3:** Comparison of the physiochemical properties of FB24 and S100A6.

Parameters	FB24	S100A6
Number of amino acids	113	91
Molecular weight	11,980.50	10,178.75
Theoretical pI	4.82	5.31
Total number of negatively charged residues (Asp + Glu)	13	15
Total number of positively charged residues (Arg + Lys)	10	12
Total number of atoms	1,656	1,452
Ext. coefficient	10,095	4,470
Estimated half-life	30 h (mammalian reticulocytes, *in vitro*). >20 h (yeast, *in vivo*) >10 h (*Escherichia coli*, *in vivo*)	30 h (mammalian reticulocytes, *in vitro*) >20 h (yeast, *in vivo*) >10 h (*Escherichia coli*, *in vivo*)
Instability index (II)	40.15	26.96
Aliphatic index	63.98	106.26
Grand average of hydropathicity (GRAVY)	−0.151	−0.232

### 3.6 Prediction and validation of the isolated VHH 3D structure

**FIGURE 3:**
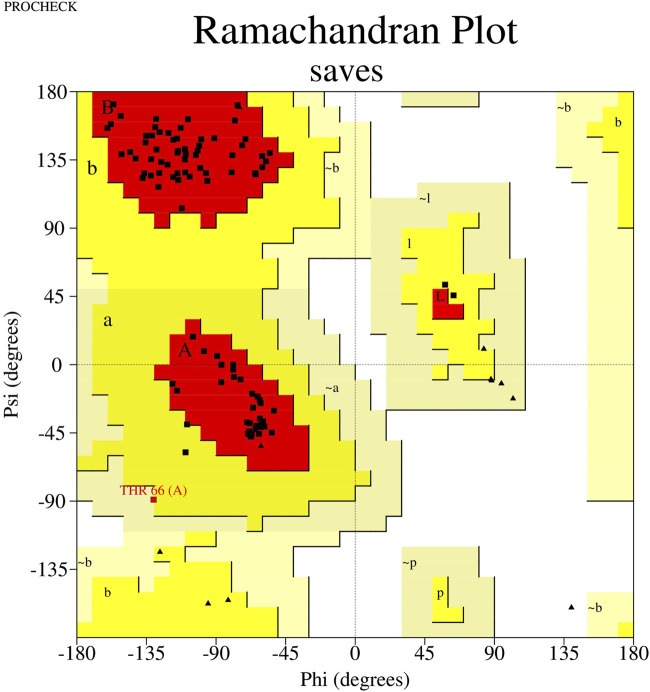
Ramachandran plot of FB24. This plot shows that 71.4% of residues are located in the favored region, 24.4% of residues are in the allowed region, and just 4.1% of residues are present in the disallowed region.

To predict FB24’s 3D model, we used the trRosetta (Du et al., 2021), I-TASSER (Yang et al., 2015), and RaptorX (Wang et al., 2017) online web servers. The template modeling score (TM-score), a metric used to evaluate the similarity between a target and template structure (and also used to evaluate model quality), was obtained for the trRosetta, I-TASSER, and RaptorX predicted 3D structure models. The TM-scores of FB24 for the trRosetta, I-TASSER, and RaptorX models were 0.792, 0.655, and 0.715, respectively. Based on the TM-scores of the models, the tr-Rosetta model demonstrated better quality than those obtained using the other prediction servers. Furthermore, assessment of the predicted models using the PROCHECK (Laskowski et al., 1996) and Verify3D (Lüthy et al., 1992) modules of SAVES (Bowie et al., 1991) web server demonstrated that the trRosetta-predicted model involves validated parameters compared to other predicted models (Supplementary Table S2). PROCHECK results of the trRosetta-predicted model showed that 95.9% of residues are situated in the favored region, 3.1% in the allowed region, and only 1.0% in the disallowed region (Figure 3; Supplementary Table S2), while just 86.7% and 71.4% residues of RaptorX and I-TASSER predicted models located in most favored regions. In addition, Verify3D module results, which provides an analysis of the compatibility of the 3D models with their amino acid sequence (1D), indicated that 78.76%, 77.56%, and 77.11% residues of trRosetta, RaptorX, and I-TASSER predicted models have averaged 3D-1D score≥0.1 (Supplementary Table S2).

### 3.7 Peptide-receptor docking and virtual screening

The docking process was performed using the HADDOCK web server that presents various data about protein-protein docking, which contains: HADDOCK score, Cluster size, RMSD from the overall lowest-energy structure, Van der Waals energy, Electrostatic energy, Desolvation energy, Restraints violation energy, Buried Surface Area, and z-score, demonstrated in Table 4. We first investigated the HADDOCK score to identify the models with the most favorable interaction energies. After that, we used the Z-score to assess these models’ relative quality and uniqueness within the context of our specific docking run. HADDOCK score is a weighted sum of different energy terms, including van der Waals energy, electrostatic energy, desolvation energy, and restraints violation energy, and its lower score generally indicates a better model because it suggests a more favorable interaction between the proteins. The docking results demonstrated that the HADDOCK score of FB24-receptors docked complexes are −33.5 ± 10.0, 35.7 ± 16.8, 15.4 ± 17.4, and 23.7 ± 13.8 which relate to FB24-RAGE, FB24-IGF-1R, FB24-TFR1, and FB24-LRP1, respectively. These scores for receptors-crystal ligands docked complexes ranged from −82.8 to −8.0 (S100A6-RAGE = −50.8 ± 10.5; IGF1-IGF-16 = −8.0 ± 12.7; 3DS18-TFR1 = −17.5 ± 5.7; and PCSK9-LRP1 = −82.8 ± 4.9) (Table 4).

**TABLE 4:** Peptide-Protein docking of FB24 and crystal ligands with ET receptors using HADDOCK online web server results.

Receptor	PDBID	Ligand	HADDOCK score	Cluster size	Z-Score	Van der Waals energy	Electrostatic energy	No. of hydrogen bonds
RAGE ectodomain (fragment VC1C2)	4P2Y	FB24	−33.5 ± 10.0	12	−2.6	−112.0 ± 1.0	−280.8 ± 20.2	18
	4P2Y	S100A6	−50.8 ± 10.5	6	−2.2	−63.0 ± 5.9	−373.1 ± 51.0	11
IGF-1R (Ectodomain)	5U8Q	FB24	35.7 ± 16.8	5	−1.7	−93.1 ± 6.7	−358.9 ± 67.5	11
	5U8Q	IGF-1	−8.0 ± 12.7	4	−1.7	−53.1 ± 8.5	−179.1 ± 19.4	7
TFR1 (Ectodomain)	6WRV	FB24	15.4 ± 17.4	19	−2.1	−68.4 ± 3.6	−307.0 ± 44.1	10
	6WRV	3DS18	−17.5 ± 5.7	16	−2.0	−38.3 ± 6.1	−353.8 ± 32.1	8
LRP1 (Ectodomain)	3M0C	FB24	23.7 ± 13.8	11	−2.7	−76.6 ± 7.0	−202.0 ± 25.0	12
	3M0C	PCSK9	−82.8 ± 4.9	12	−2.1	−39.3 ± 4.9	−236.7 ± 9.0	5

The z-score is a statistical measurement that exhibits how many standard deviations a score is from the mean score of the generated models. It helps to assess the relative quality of a docking complex within the context of a particular docking run. A more negative Z-score means the model is better in the context of the other generated models. The z scores for FB24-receptors ranged from −1.7 to −2.7, whereas crystal ligands-receptors ranged from −1.7 to −2.2. Additionally, the electrostatics and van der Waals interactions of FB24-receptors displayed values within specified ranges (Table 4). The results showed that the z-score of FB24-IGF-1R and FB24-IGF1 is the same (−1, 7), and the scores for FB24-TFR1 and 3DS18-TFR1 are very close together (−2.1 and −2.0, respectively). But FB24-IGF-1R vs FB24-IGF1 and FB24-TFR1 vs 3DS18-TFR1 HADDOCK scores are very different. This is because the HADDOCK score is the sum of various calculated energies, which vary between FB24-receptor and their respective crystal ligand-receptor complexes. Meanwhile, the z-score shows the standard deviations of a score from the mean score of the generated models that are the same or very close between FB24-receptor and their respective crystal ligand-receptor complexes.

**FIGURE 4:**
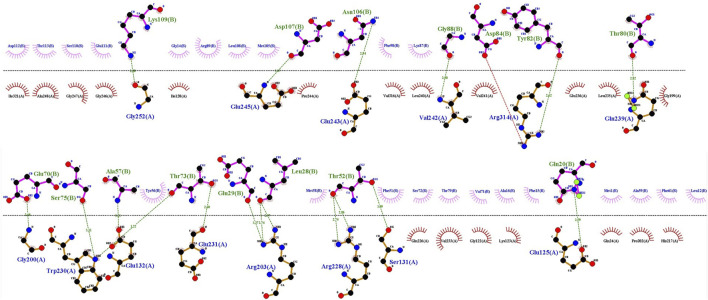
FB24-RAGE bonds visualization using LigPlot. Hydrogen bond: green dotted line; non-bonded involved residues of RAGE and FB24 shown in red and pink, respectively.

**FIGURE 5:**
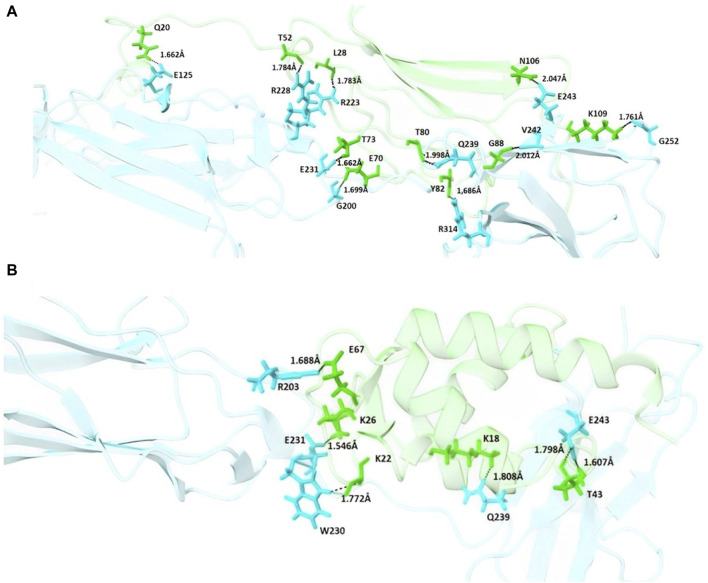
Schematic interaction of FB24-RAGE and S100A6-RAGE complexes. **(A)** Residues of the FB24-RAGE complex involved in hydrogen bonding interactions are shown in sticks, with the rest of the structures shown in cartoon; **(B)** Residues of the S100A6-RAGE complex involved in hydrogen bonding interactions are shown in sticks, with the rest of the structures shown in cartoon. In each complex, residues of the ligands, including FB24 and S100A6, are shown in green, and the residues of the receptor are shown in blue.

In addition to the mentioned analysis, the protein-protein docking results were further evaluated through PRODIGY HADDOCK (Supplementary Table S3) and PDBsum (Supplementary Table S4). By comparing the docking results of FB24 with the crystal ligand structures, it was observed that FB24-receptor interactions exhibited superior hydrogen bond counts and binding affinity compared to crystal ligands. Overall, FB24-RAGE demonstrated a lower HADDOCK score (−33.5 ± 10.0) and z-score (−2.6), and also, this docked complex showed higher binding affinity and more hydrogen bonds compared with FB24 complexes with other receptors (Table 4). The number of hydrogen bonds and non-bonded interactions analysis of BF24-RAGE by LigPlot software (Pettersen et al., 2004) showed 18 hydrogen bonds and more than 20 hydrophobic interactions (Figure 4). In addition, hydrogen bonds of FB24-RAGE and S100A6-RAGE complexes are compared using Chimera 1.15src software (Wallace et al., 1995) and presented in Figures 5A, B, respectively. This analysis shows comparable interactions of FB24 with the RAGE compared with S100A6. So, we assumed that the RAGE receptor could be a potential receptor for transferring FB24 across the BBB, and we selected FB24-RAGE and S100A6-RAGE complexes for more analysis using MD simulation.

### 3.8 Molecular dynamics (MD) simulations

Based on the results of peptide-protein docking analysis, it was discovered that FB24 has a stronger bond with the RAGE V-domain compared to its crystal ligand (S100A6). After selecting the most favorable docked complexes, the physicochemical properties of the S100A6 ligand were predicted using the protparam online server (https://web.expasy.org/protparam/) (Gasteiger et al., 2005; Walker, 2005) and compared with FB24. The analysis results showed that the half-life of S100A6 in mammals is similar to that of FB24, but its instability index is 26.96, which is smaller than FB24’s instability index of 40.15. Detailed physicochemical characteristics of S100A6 and FB24 are provided in Table 3.

Additionally, to assess the stability of the FB24-RAGE and compare it with S100A6-RAGE, a 500 ns MD simulation was conducted using GROMACS. The analysis of the MD simulation was carried out utilizing GROMACS internal modules and visualized by OriginLab software (OriginPro, 2016).

**FIGURE 6:**
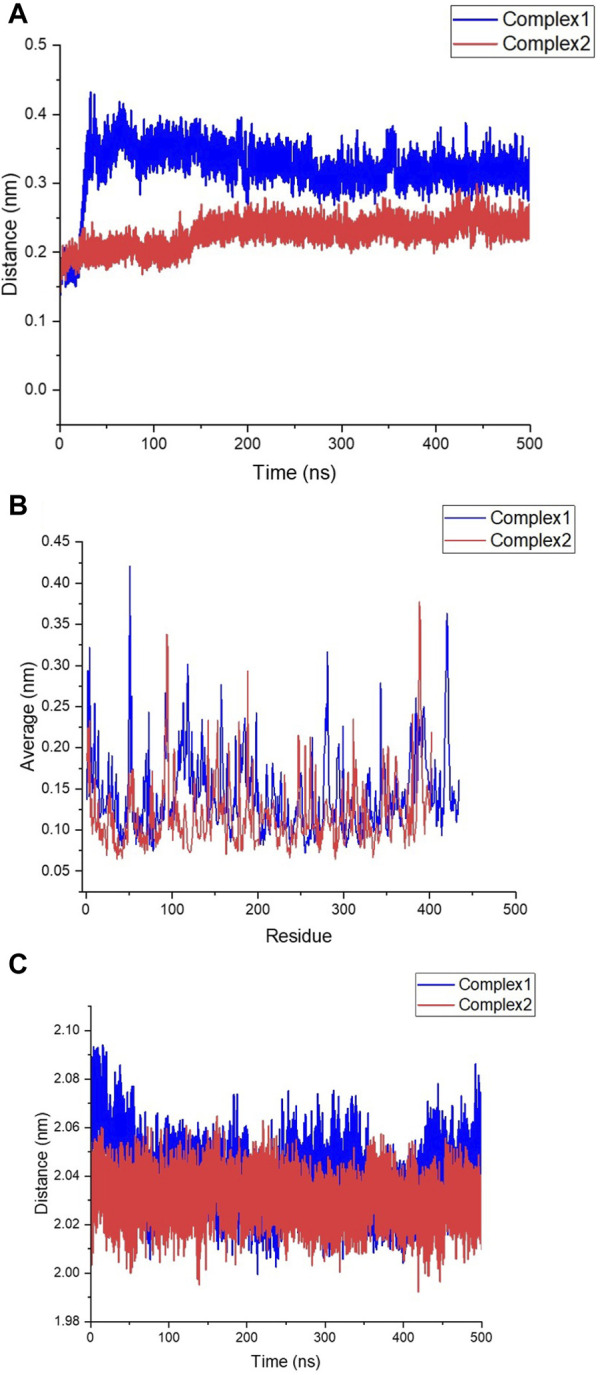
Stability assessment of FB24-RAGE (Complex1) and S100A6-RAGE (Complex2). **(A)** Root Mean square Deviation (RMSD) of Complex1 and Complex2 structures during 500 ns molecular dynamics (MD) simulation. **(B)** Root Mean square fluctuation (RMSF) of Complex1 and Complex2 residues during 500 ns MD simulation. **(C)** Radius of gyration (RG) of Complex1 and Complex2 structures during 500 ns MD simulation; Complex1 and Complex2 shown in blue and red, respectively.

The deviation and fluctuation of Complex1 (FB24-RAGE) and Complex2 (S100A6-RAGE) are illustrated in Figures 6A, B in red and blue, respectively. The mean RMSD value at the equilibrated state was 0.320 nm for Complex1 and 0.226 nm for Complex2. The Complex1 revealed a significant increasing deviation at 40 ns, and after that, the Complex1 deviation decreased and reached a more stable state after 240 ns with a minor deviation. However, the Complex2 showed insignificant deviations from its basic structure, and an increasing deviation of the Complex2 revealed about 150 ns after that, its structure reached a stable structure with minor deviation. Complex1 displayed more variation and deviation during the simulation until the equilibrium state was reached. Conversely, Complex2 showed fewer changes, with the value remaining constant after 150 ns

The RMSF plot of residues depicted fluctuations in the loop structures throughout the simulation time. Comparing Figures 6A, B, it is evident that Complex1 had more fluctuations in its peaks compared to Complex2. The compactness of the structures was assessed by measuring the radius of gyration (Rg). Figure 6C illustrates that both Complex1 and Complex2 maintained stability with Rg values of 2.040 nm and 2.030 nm, respectively.

Despite the worse metrics for FB24 relative to S100A6, they are still generally comparable, and allow us to suggest that FB24 could be a potential binder to the RAGE receptor and could be a potential candidate for further investigation in theranostic compound delivery to the brain as a conjugated sdAb on the surface of vehicles.

## 4 Discussion

The challenge of delivering drugs to the brain remains a significant obstacle for researchers and physicians in the treatment of brain disorders. Various methods, such as physically damaging the BBB, have been attempted in the past to overcome this limitation. However, these approaches often led to undesirable side effects, prompting scientists to explore alternative ways to deliver compounds to the brain while minimizing harm to the BBB.

As a result, nanocarriers have emerged as a promising new method for drug delivery to the brain (Hasannejad-Asl et al., 2023). Among these nanocarriers, quantum dots (QDs) have shown potential for delivering drugs to treat mental disorders and brain diseases such as Alzheimer’s disease (AD) and glioblastoma (GB) (Hasannejadasl et al., 2020). In recent years, the use of diverse nanometric and polymeric carriers, as well as single-domain antibodies (sdAbs), has gained attention as biotherapeutic agents and targeted delivery systems for crossing the BBB. Peptides, antibodies, and VHH ligands have been developed to target specific endocytosing BBB receptors, acting as molecular Trojan horses or shuttles to transport therapeutic cargo across the BBB.

Researchers have conducted molecular studies and antibody library screenings to identify new pairs of receptor-mediated transcytosis (RMT) antibodies for delivering drugs across the BBB (Darmanis et al., 2015; Uchida et al., 2015; He et al., 2018; Vanlandewijck et al., 2018). For example, Wael Alata and colleagues (Alata et al., 2022) designed three sdAbs (IGF1R3, IGF1R4, and IGF1R5) from immunized llamas against the IGF1R receptor, resulting in enhanced transmigration across the BBB in a cell culture model. By utilizing a naïve camelid PBMC library, researchers were able to generate a vast pool of VHHs to target various receptors efficiently.

Through amplification of VHH cDNA recovered from *camelus dromedarius* PBMCs using nested PCR, a comprehensive nanobody library was established for identifying potential drug carriers. By employing multiple primer pairs, the library repertoire was expanded to encompass a wide range of possibilities for targeted drug delivery. Initial studies utilizing PD libraries of VHHs identified FC5 and FC44 as powerful sdAbs capable of crossing the BBB and accessing the brain with high accumulation, while being rapidly cleared from the kidneys and liver in mice. These two VHHs showed considerable brain accumulation and fast kidney and liver clearance in mice (Muruganandam et al., 2002).

FC5 was found to interact with active RMT through the luminal alpha (Pardridge, 2017; Hasannejad-Asl et al., 2023)-sialoglycoprotein receptor (TMEM30A) (Abulrob et al., 2005) and was then modified into FC5-Fc-neuropeptide for confirmation of its ability to enter the central nervous system (Farrington et al., 2014; Webster et al., 2016). Our FB24, obtained from brain tissue, contains over 70% of these single-domain antibodies. By using tween-20 for perfusion in our research, we determined that FB24 was not attached to endothelial cells and was sourced from brain tissue.

*In vitro* testing, which occurs in a laboratory setting, involves the study of microorganisms, human cells, or animal cells in culture. This allows scientists to investigate various biological processes within individual cells without outside influences or complexities found in whole organisms. While *in vitro* models do not fully replicate the complex structure of the brain, they serve as a useful tool for understanding biological mechanisms. To address this limitation, we conducted *in vivo* screening to identify stable VHH that could effectively cross the blood-brain barrier in physiological conditions.

Utilizing *in vivo* panning and perfusion techniques with normal saline containing tween-20, we have strong evidence to suggest that FB24 can effectively cross the BBB and enter the brain. Given its macromolecular nature, FB24 must utilize cell surface receptors in the RMT pathway to reach the brain. The binding of FB24 to various receptors was explored through molecular docking with the most prevalent receptors: TfR, IGFR, LDLR, and RAGE.

Categorized by their functionality, endothelial cell transporters involved in blood-brain barrier crossing include iron transporters (TfR) (Gutiérrez et al., 2016; Pardridge, 2017; Honorato et al., 2021), insulin and insulin-like growth factors receptors (INSR, IGF1R, IGF2R) (OriginPro, 2016; Vanlandewijck et al., 2018), lipid transporters (LDLR, LRP1, LRP8, TMEM30A/CDC50A) (Darmanis et al., 2015; Uchida et al., 2015; He et al., 2018; Alata et al., 2022), solute carrier family transporters (GLUT-1/SLC2A1, SLC3A2/CD98hc) (Muruganandam et al., 2002; Abulrob et al., 2005), and neuropeptide receptors (LEPR) (Liu et al., 2010). The receptor for advanced glycation end products (RAGE) has been identified as playing a crucial role in Aβ binding in neurons, microglia, and the blood-brain barrier (Liu et al., 2010; Farrington et al., 2014; Webster et al., 2016). Aβ binds to RAGE primarily through its extracellular V domain.

In our analysis, FB24 demonstrated a higher affinity towards the receptors compared to their crystal structures, with the strongest interaction observed with the RAGE receptor. Studies have shown specific interaction interfaces between RAGE and Aβ isoforms, further supporting the potential for FB24 to effectively bind to these critical interaction sites. Through docking analysis, the study identified two interfaces: one characterized by an extended area at the junction of RAGE domains V and C1, and the other by a smaller area linking C1 and C2 domains. Considering that RAGE is applied for Aβ and other peptide transfer and FB24 strongly binds to key interaction interfaces of RAGE, it can be argued that our predicted path can be reasonable. MD simulations confirmed the stability of FB24 binding to RAGE, reinforcing the likelihood of this predicted pathway.

In general, FB24 is described as a BBB-penetrating nanobody that can serve as a carrier for delivering therapeutic substances to the brain. However, further research is required to determine its precise localization in the brain and the specific molecular mechanism it utilizes to penetrate the blood-brain barrier. Following the validation of FB24’s ability to transfer across the BBB, this nanobody needs to be conjugated on the surface of other vehicles, such as QDs and lipid nanoparticles, which carry theranostic contents, to investigate its potential to increase efficiency.

## 5 Conclusion

In conclusion, central nervous system (CNS) disorders pose a significant health challenge due to the BBB impeding their treatment. Therefore, it is essential to identify carriers capable of crossing the BBB without compromising this protective barrier that blocks harmful agents from entering the brain while facilitating the delivery of therapeutic compounds to the CNS. Recently, in addition to traditional antibodies and their derivatives, single-domain antibodies known as VHHs have emerged as promising candidates for the treatment, diagnosis, and transportation of drugs.

In this research, a VHH named FB24 was extracted from the brain of an animal model and its potential mechanisms were investigated through the receptor-mediated transcytosis (RMT) pathway. Despite FB24 demonstrating a strong affinity for endothelial cells (ECs) receptors, particularly RAGE, compared to their crystal ligand, further analysis is necessary to ascertain its migration mechanism and localization within the brain.

## Data Availability

The data presented in the study are deposited in the Nucleotide repository, accession number MK359289 (https://www.ncbi.nlm.nih.gov/nuccore/MK359289).
