# Supplementary material for: Molecular dynamics simulation of the brain-isolated single-domain antibody/nanobody from camels through in vivo phage display screening
Source: Front Mol Biosci. 2024 Sep 2;11:1414119. doi: 10.3389/fmolb.2024.1414119 (PMC11406554; doi:10.3389/fmolb.2024.1414119)
Supplement: Supplementary file 1 [file Table1.docx]

**SUPPLEMENTARY TABLE S1**: 3D structures of FB24 and EC receptors as well as their crystal structure ligands. Β-sheet, α-helix, and coil shown in yellow, red, and green, respectively.

| **Protein** | **Crystal structure** |
| --- | --- |
| **Isolated FB24 predicted structure** | |
| FB24 | 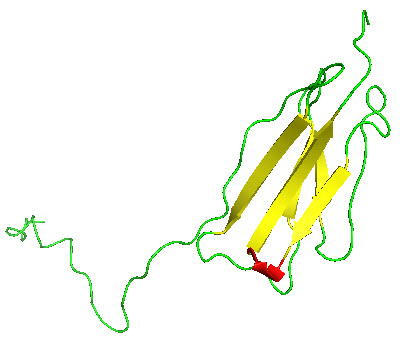 |
| **Crystal receptor’s structure** | |
| RAGE | 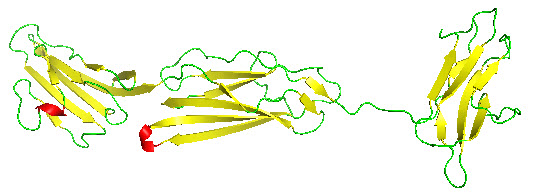 |
| TFR1 | 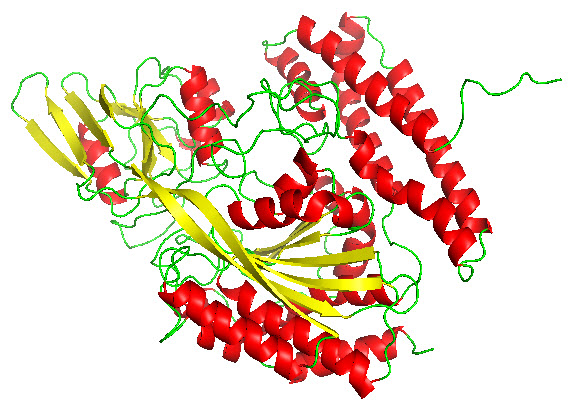 |
| LRP1 | 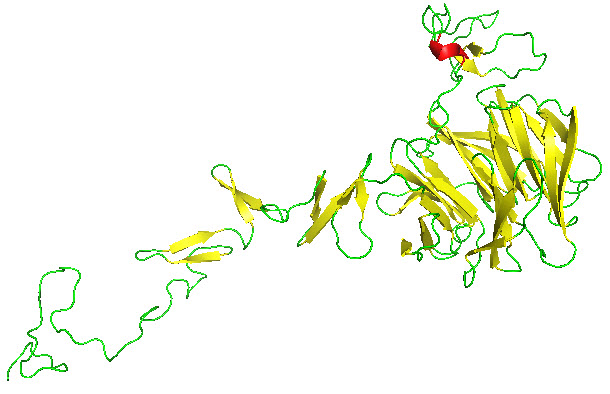 |
| IGF-1R | 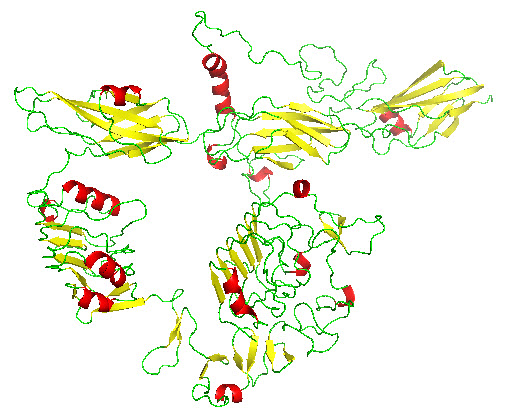 |
| **Crystal ligand’s structure** | |
| S100A6 | 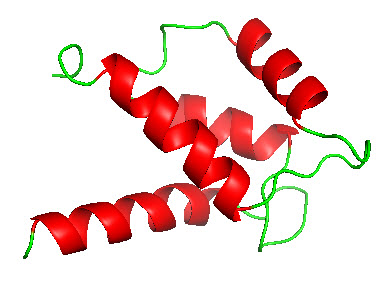 |
| 3DS118 | 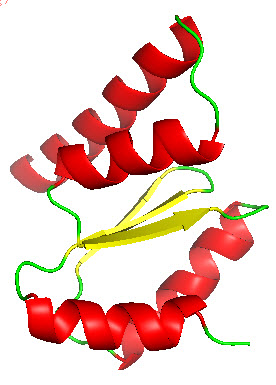 |
| PCSK9 | 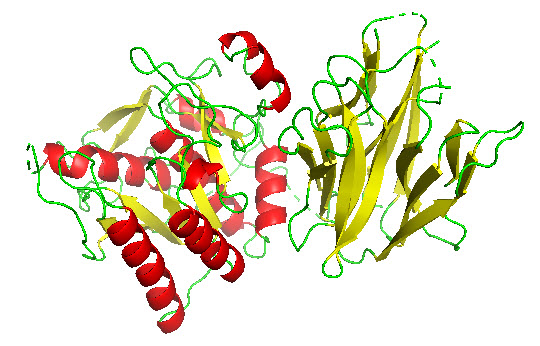 |
| IGF-1 | 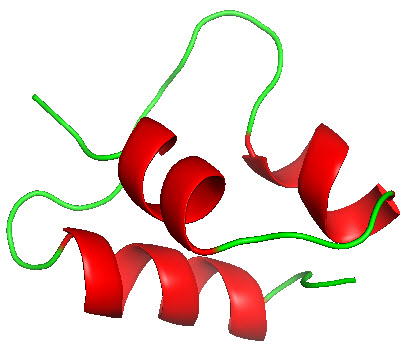 |
