# Supplementary material for: Molecular dynamics simulation of the brain-isolated single-domain antibody/nanobody from camels through in vivo phage display screening
Source: Front Mol Biosci. 2024 Sep 2;11:1414119. doi: 10.3389/fmolb.2024.1414119 (PMC11406554; doi:10.3389/fmolb.2024.1414119)

**SUPPLEMENTARY TABLE S2**: Analysis of predicted 3D structure models by Verify3D and PROCHECK modules of SAVES online web servers.

| **3D model prediction server** | **Verify3D analysis** | **PROCHECK analysis** | **RMSD** |
| --- | --- | --- | --- |
| **trRosetta** | 78.76% of the residues have  averaged 3D-1D score >= 0.1 | Residues in most favored regions [A,B,L] 94 95.9%  Residues in additional allowed regions [a,b,l,p] 3 3.1%  Residues in generously allowed regions [~a,~b,~l,~p] 1 1.0%  Residues in disallowed regions 0 0.0%  ---- ------  Number of non-glycine and non-proline residues 98 100.0%  Number of end-residues (excl. Gly and Pro) 2  Number of glycine residues (shown as triangles) 11  Number of proline residues 2  ----  Total number of residues 113 | TM-score= 0.792 |
| **I-TASSER** | 77.11% of the residues have  averaged 3D-1D score >= 0.1 | Residues in most favored regions [A,B,L] 70 71.4%  Residues in additional allowed regions [a,b,l,p] 22 22.4%  Residues in generously allowed regions [~a,~b,~l,~p] 2 2.0%  Residues in disallowed regions 4 4.1%  ---- ------  Number of non-glycine and non-proline residues 98 100.0%  Number of end-residues (excl. Gly and Pro) 2  Number of glycine residues (shown as triangles) 11  Number of proline residues 2  ----  Total number of residues 113 | TM-score= 0.655 |
| **RaptorX** | 77.56% of the residues have  averaged 3D-1D score >= 0.1 | Residues in most favored regions [A,B,L] 85 86.7%  Residues in additional allowed regions [a,b,l,p] 13 13.3%  Residues in generously allowed regions [~a,~b,~l,~p] 0 0.0%  Residues in disallowed regions 0 0.0%  ---- ------  Number of non-glycine and non-proline residues 98 100.0%  Number of end-residues (excl. Gly and Pro) 2  Number of glycine residues (shown as triangles) 11  Number of proline residues 2  ----  Total number of residues 113 | TM-score= 0.715 |

**Abbreviation**: TM-score: Template modeling score

**Visualization of Verify3D analysis**


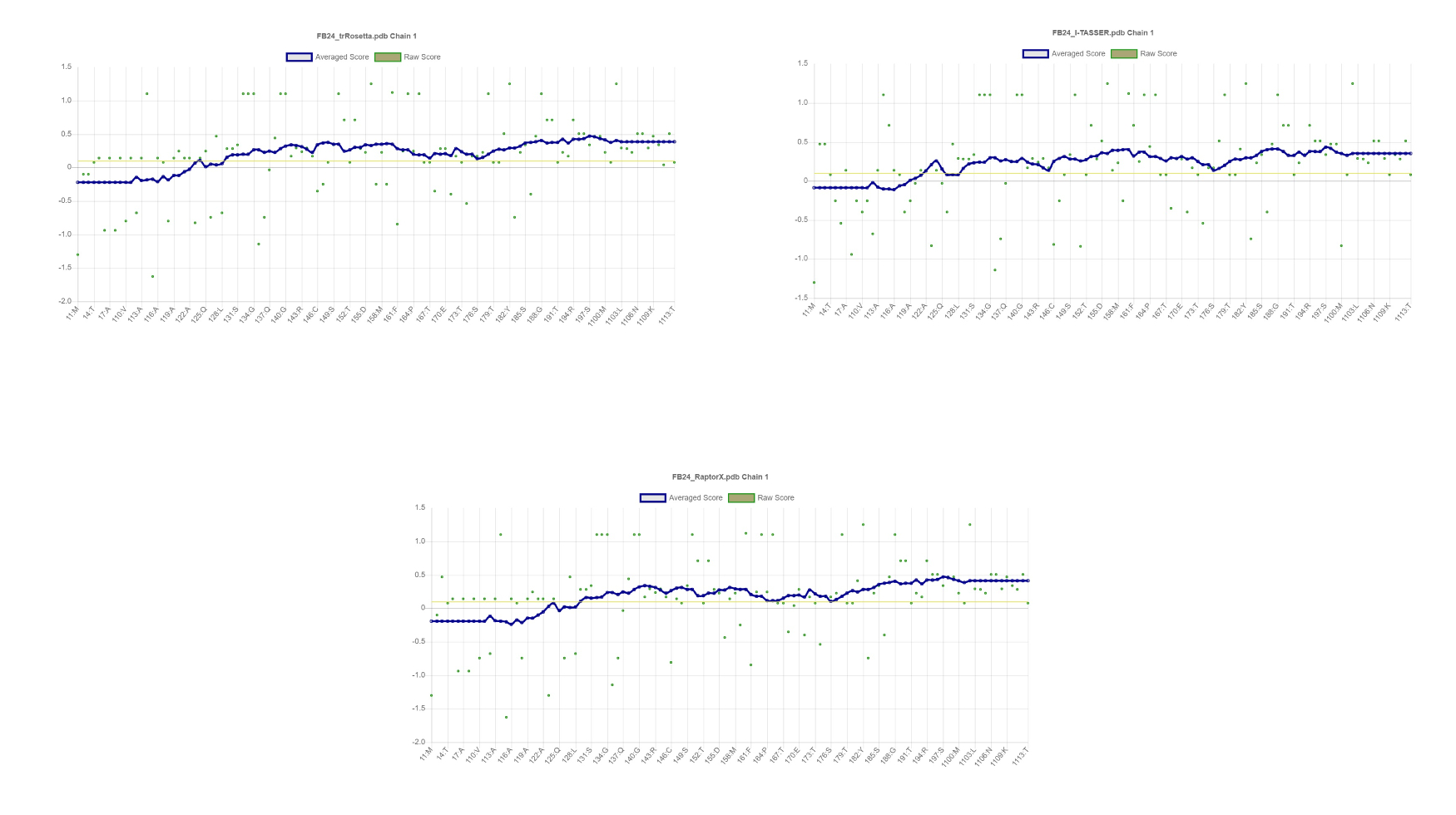


**Visualization of PROCHECK analysis resulted in Ramachandran plot**


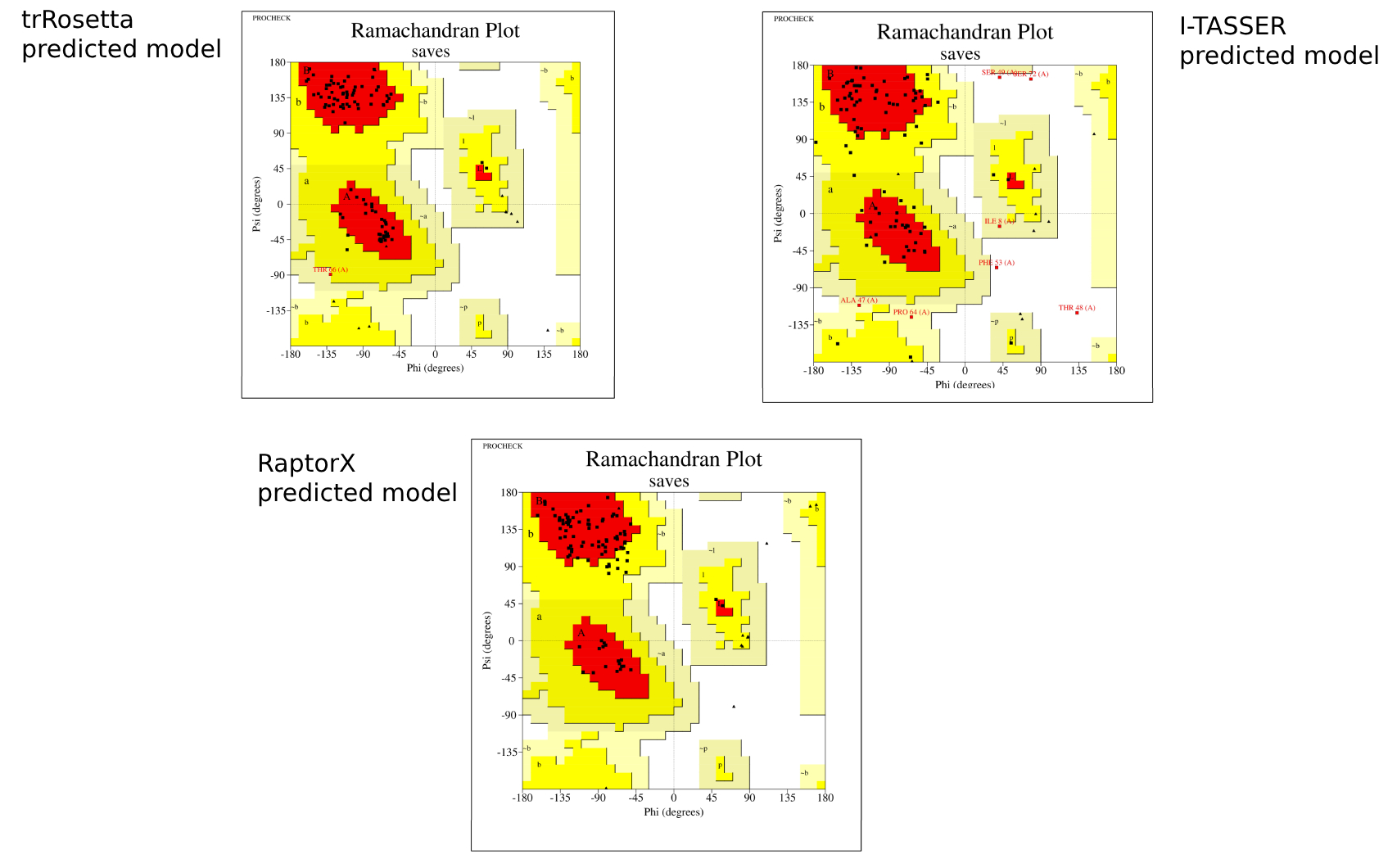

Supplement: Supplementary file 2 [file Table2.docx]
