# Supplementary material for: Molecular dynamics simulation of the brain-isolated single-domain antibody/nanobody from camels through in vivo phage display screening
Source: Front Mol Biosci. 2024 Sep 2;11:1414119. doi: 10.3389/fmolb.2024.1414119 (PMC11406554; doi:10.3389/fmolb.2024.1414119)
Supplement: Supplementary file 3 [file Table3.docx]

**SUPPLEMENTARY TABLE S3**: Binding affinity and Kd prediction using PRODIGY server.

| **Protein-protein complex** | **ΔG (kcal mol-1)** | **Kd (M) at ℃** | **ICs charged-charged** | **ICs charged-polar** | **ICs charged-apolar** | **ICs polar-polar** | **ICs polar-apolar** | **ICs apolar-apolar** | **NIS charged** | **NIS apolar** |
| --- | --- | --- | --- | --- | --- | --- | --- | --- | --- | --- |
| **FB24-RAGE** | -16.9 | 4.1e-13 ℃ | 13 | 14 | 58 | 5 | 32 | 32 | 22.13 | 49.8 |
| **S100A6-RAGE** | -11.2 | 6e-09 ℃ | 16 | 11 | 402 | 2 | 13 | 34 | 24.46 | 5.46 |
| **FB24- IGF-R1** | -12.5 | 6.9e-10 ℃ | 18 | 13 | 20 | 5 | 20 | 38 | 27.13 | 37.31 |
| **IGF-1- IGF-R1** | -13.0 | 3e-10 ℃ | 3 | 8 | 23 | 5 | 27 | 23 | 27.59 | 36.84 |
| **FB24-TFR1** | -12.6 | 5.7e-10 ℃ | 12 | 12 | 30 | 4 | 18 | 22 | 27.54 | 37.32 |
| **3DS118-TFR1** | -7.6 | 2.5e-06 ℃ | 6 | 10 | 15 | 3 | 5 | 23 | 32.76 | 34.3 |
| **FB24-LRP1** | -12 | 1.7e-09 ℃ | 8 | 10 | 24 | 5 | 22 | 22 | 24.95 | 41.29 |
| **PCSK9-LRP1** | -9 | 2.4e-07 ℃ | 5 | 4 | 12 | 4 | 14 | 16 | 25.14 | 40.23 |

**Columns description (1):**

- Predicted value of the binding affinity (ΔG) expressed in kcal mol-1
- Calculated value of the dissociation constant (Kd) at a given temperature (25 ℃ by default), expressed in Molar (M)
- Number of intermolecular contacts (ICs) at the interface within the threshold distance of 5.5 Å, separately listed according to the contact property
- Percentage of the charged and apolar non-interacting surface (NIS%) of the complex

1. Honorato RV, Koukos PI, Jiménez-García B, Tsaregorodtsev A, Verlato M, Giachetti A, et al. Structural biology in the clouds: the WeNMR-EOSC ecosystem. Frontiers in molecular biosciences. 2021;8:729513.
