# Supplementary material for: Molecular dynamics simulation of the brain-isolated single-domain antibody/nanobody from camels through in vivo phage display screening
Source: Front Mol Biosci. 2024 Sep 2;11:1414119. doi: 10.3389/fmolb.2024.1414119 (PMC11406554; doi:10.3389/fmolb.2024.1414119)
Supplement: Supplementary file 4 [file Table4.docx]

**SUPPLEMENTARY TABLE S4:** HADDOCK’s result analysis using PDBsum

| **Chain** | **No. of  interface  residues** | **Interface   area (Å2)** | **No. of salt bridges** | **No. of  disulphide  bonds** | **No. of  hydrogen  bonds** | **No. of  non-bonded   contacts** |
| --- | --- | --- | --- | --- | --- | --- |
| **RAGE – BF-24 docking in S100A6 binding residues** | | | | | | |
| **RAGE**  **FB-24** | 34  38 | 1863  1674 | 1 | - | 18 | 213 |
| **RAGE**  **S100A6** | 28  31 | 1431  1369 | 4 | - | 11 | 166 |
| **IGF-1R – BF-24 and IGF-1R-IGF1 docking in IGF1 binding residues** | | | | | | |
| **IGF-1R**  **FB-24** | 30  33 | 1559  1544 | 5 | - | 11 | 143 |
| **IGF-1R**  **IGF1** | 17  24 | 979  878 | 1 | - | 7 | 158 |
| **TFR1 – BF-24 docking in PCSK9 binding residues** | | | | | | |
| **TFR1**  **FB-24** | 27  23 | 1218  1223 | 2 | - | 10 | 139 |
| **TFR1**  **3DS118** | 14  19 | 818  754 | 4 | - | 8 | 68 |
| **LRP1 – BF-24 docking in 3DS18 binding residues** | | | | | | |
| **LRP1**  **FB-24** | 24  29 | 1132  966 | 1 | - | 12 | 173 |
| **LRP1**  **PCSK9** | 12  11 | 637  635 | 2 | - | 5 | 61 |
